# Characterization of Three Novel Papillomavirus Genomes in Vampire Bats (*Desmodus rotundus*)

**DOI:** 10.3390/ani14243604

**Published:** 2024-12-14

**Authors:** Laura Junqueira de Camargo, Raquel Silva Alves, Raíssa Nunes dos Santos, Letícia Ferreira Baumbach, Juliana do Canto Olegário, Vitória Rabaioli, Matheus de Oliveira Silva, André Alberto Witt, Fernanda Marques Godinho, Richard Steiner Salvato, Matheus Nunes Weber, Mariana Soares da Silva, Cíntia Daudt, Renata da Fontoura Budaszewski, Cláudio Wageck Canal

**Affiliations:** 1 Laboratório de Virologia Veterinária, Faculdade de Veterinária, Universidade Federal do Rio Grande do Sul (UFRGS), Porto Alegre 91540-000, Brazil; laurajcamargo@gmail.com (L.J.d.C.); raquels.alves@hotmail.com (R.S.A.); leticiabaumbach@yahoo.com.br (L.F.B.); juli.ole@hotmail.com (J.d.C.O.); vitoria.rabaioli1@gmail.com (V.R.); mdeoliveira344@gmail.com (M.d.O.S.); rebud@hotmail.com (R.d.F.B.); claudio.canal@ufrgs.br (C.W.C.); 2 Laboratório de Virologia, Instituto de Ciências Básicas da Saúde (ICBS), Universidade Federal do Rio Grande do Sul (UFRGS), Porto Alegre 90035-003, Brazil; engraissanunes@gmail.com; 3 Secretaria Estadual de Agricultura, Pecuária e Desenvolvimento Rural (SEAPDR), Rio Grande do Sul, Porto Alegre 90150-004, Brazil; andrewitt15@gmail.com; 4 Centro Estatual de Vigilância em Saúde (CEVS)—Centro de Desenvolvimento Científico e Tecnológico (CDCT), Rio Grande do Sul, Porto Alegre 90450-190, Brazil; fernandamsg7@gmail.com (F.M.G.); richardsalvato@hotmail.com (R.S.S.); 5 Laboratório de Imunologia e Biologia Molecular, Faculdade de Veterinária, Universidade Federal do Rio Grande do Sul (UFRGS), Porto Alegre 91540-000, Brazil; 6 Laboratório de Microbiologia Molecular, Instituto de Ciências da Saúde, Universidade Feevale, Novo Hamburgo 93525-075, Brazil; mariana2@feevale.br; 7 Laboratório de Virologia Geral e Parasitologia (LABVIRPA), Universidade Federal do Acre (UFAC), Rio Branco 69920-900, Brazil; cintia.daudt@ufac.br

**Keywords:** bats, haematophagous, novel viruses, *Papillomaviridae*, sequencing

## Abstract

Bats are mammals with high biodiversity and wide geographical range. In Brazil, three haematophagous bat species are found. *Desmodus rotundus* is the most documented due to its role as a primary host of rabies virus in Latin America. Bats are known to harbor various emerging viruses causing severe human diseases. Beyond zoonotic viruses, these animals also harbor a diversity of non-zoonotic viruses. Papillomaviruses are circular double-stranded deoxyribonucleic acid (dsDNA) viruses that infect the epithelial and mucosal cells of many vertebrates, occasionally causing malignant lesions. High-throughput sequencing has enabled papillomaviruses discovery in different bat species. Here, 22 *D. rotundus* samples were collected through the rabies eradication program in Rio Grande do Sul. The DNA extracted from pooled intestines was amplified by the rolling-circle amplification (RCA) method and sequenced using the Illumina^®^ MiSeq platform (San Diego, CA, USA).Analysis revealed three contigs corresponding to the *Papillomaviridae* family, representing three novel viruses named DrPV-1, DrPV-2, and DrPV-3. Phylogenetic analysis suggests DrPV-1 may constitute a new species within the *Dyophipapillomavirus* genus, while DrPV-2 and DrPV-3 may represent different types within the same species from a novel genus. This is the first description of a papillomavirus in the *D. rotundus* species, contributing to the characterization of PVs in the Chiropteran order.

## Simple Summary

Bats represent a vast number of species, and Chiroptera is the second most diverse order of mammals. *Desmodus rotundus*, or the common vampire bat, is the most studied haematophagous species, as it is the primary host of the rabies virus in Latin America. Through high-throughput sequencing, we identified and characterized three novel members and a potential new genus of the *Papillomaviridae* family, named DrPV-1, DrPV-2, and DrPV-3. Papillomaviruses are capable of infecting many vertebrates, including bats, and can occasionally cause cancer. These findings contribute to the characterization of papillomaviruses in understudied groups such as bats, particularly in *D. rotundus*, whose viral diversity is often biased toward rabies detection. To the best of the authors’ knowledge, this is the first description of papillomaviruses in *D. rotundus*.

## 1. Introduction

The Chiroptera order is the second most diverse mammal order and comprises approximately 1400 species [1]. Bats play essential roles in maintaining ecological balance through insect control, seed dispersal, and pollination [2]. In Brazil, 182 species have been cataloged [3], including three species of haematophagous bats: the common vampire bat (*Desmodus rotundus*), the white-winged vampire bat (*Diaemus youngi*), and the hairy-legged vampire bat (*Diphylla ecaudata*) [4]. Among them, the common vampire bat is the most extensively studied species in the literature [4]. *D. rotundus* is also recognized as the primary wildlife reservoir of rabies virus in Latin America [5].

Their specialized immune system, capability for active flight, gregarious habits, living in large groups, and sharing of roosting sites with a wide variety of different bat species may explain why they are such a unique viral reservoir [6]. Various emerging viruses that cause severe diseases in humans, such as filoviruses, henipaviruses, and betacoronaviruses such as Middle East Respiratory Syndrome Coronavirus (MERS-CoV), Severe Acute Respiratory Syndrome Coronavirus (SARS-CoV), and a betacoronavirus closely related to Severe Acute Respiratory Syndrome Coronavirus 2 (SARS-CoV-2), are hosted in bats [7]. The increasing occurrence of virus spillover events from bats is multilayered and appears to result from a combination of disturbances to bat habitats, including climate change, human-driven urbanization, agricultural encroachments, demand for bushmeat, and the trafficking of exotic wildlife [8,9]. These events typically require an intermediate host to establish successful infection in humans [10]. Beyond zoonotic viruses, these animals also harbor a diversity of non-zoonotic viruses.

Papillomaviruses (PVs) are nonenveloped small viruses with circular dsDNA genomes that vary in size from 5748 to 8607 bp [11]. PVs genomes encode six to nine proteins, although the ancestral papillomavirus might consist solely of a group of core proteins: E1, E2, L1, and L2 [12]. PVs are divided into two subfamilies (*Firstpapillomavirinae* and *Secondpapillomavirinae*) with approximately 50 genera and 130 species. PVs infect the epithelial and mucosal cells of many animal hosts, such as birds, fish, reptiles, dogs, cats, bovines, and other domestic and wild mammals [12,13,14,15,16]. Most papillomavirus infections are asymptomatic, but some types are associated with lesions and even cancers [17]. Human papillomaviruses (HPVs) have more than 230 genotypes, some of which are carcinogenic and responsible for 4.5% of all human cancers [18]. PVs are known to be strictly species-specific [19]; nevertheless, exceptional cross-infections have been observed. Cats seem to be unusually prone to contracting papillomavirus infections from the HPV 9 type [20]. The feline sarcoid-associated papillomavirus (FeSarPV) was found in bovine cutaneous warts, as well as in captive African lions (*Panthera leo*) [21,22]. Bovine papillomavirus (BPV) has a well-documented history of infecting a broad range of wild ruminants [14]. The BPV1, BPV2, and BPV13 types can cause equine sarcoids [23,24], and the BPV1 type was also detected in skin lesions of a sable antelope (*Hippotragus niger*) and giraffe (*Giraffa camelopardalis*) [25].

Rolling-circle amplification (RCA) followed by high-throughput sequencing (HTS) offers a robust alternative for identifying new PVs [26]. HTS has enabled the discovery of new PVs in humans as well as wild and domestic animals [25,26,27,28,29,30,31]. Through HTS, PVs have also been detected in many bat species, such as *Miniopterus schreibersii*, *Eptesicus serotinus*, *Rhinolophus ferrumequinum*, *Eidolon serotinus*, *Eidolon isabellinus*, *Tadarida brasiliensis*, *Eidolon helvum*, *Taphozous perforatus*, *Eonycteris spelaea*, *Artibeus lituratus*, *Molossus molossus*, and *Eumops bonariensis* [32,33,34,35,36,37,38,39,40,41,42,43,44]. In this context, the present study describes the detection and characterization of three novel papillomavirus genomes obtained from vampire bats captured in southern Brazil.

## 2. Materials and Methods

### 2.1. Bat Capture and Sample Collection

**Figure 1:**
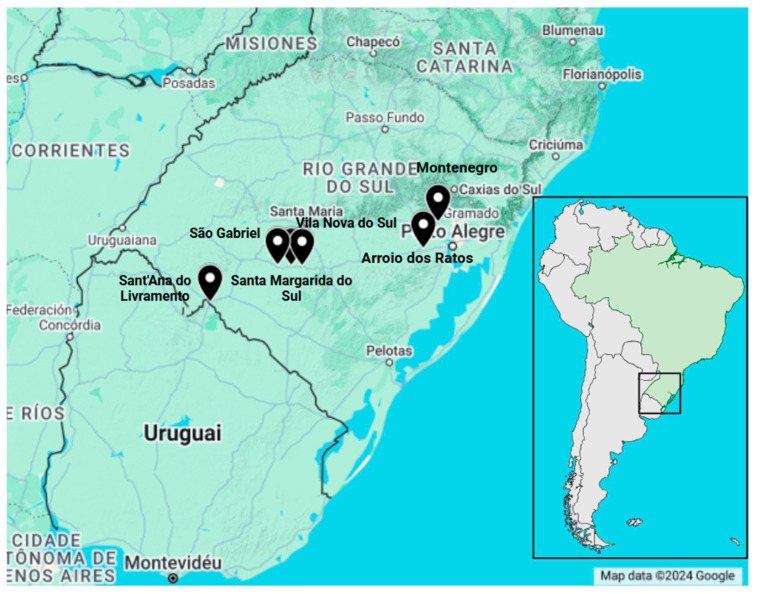
Map of the six municipalities of Rio Grande do Sul where the bat captures were conducted. The names of the municipalities are highlighted and identified with pins. De Camargo, L. (2024) https://BioRender.com/o59z907 (accessed on 15 November 2024).

Vampire bats (*Desmodus rotundus*) (*n* = 22) were captured using mist nets in Rio Grande do Sul State (southern Brazil) in the Sant’ana do Livramento, São Gabriel, Santa Margarida do Sul, Vila Nova do Sul, Arroio dos Ratos, and Montenegro municipalities (Figure 1), during August and November 2022. The captures were conducted as part of the states’ official continuous rabies surveillance program [45]. The map (Figure 1) was created using BioRender and google maps.

The project was authorized by ICMBio (Instituto Chico Mendes de Conservação da Biodiversidade) under protocol number #89992-1. Bats were euthanized using an overdose of anesthetic ketamine (1 mL) injected in the ventral region of the animal, following the recommendations of Resolutions CFMV No. 1000/2012 and CFBio No. 301/2012. During necropsy, intestines containing feces were collected and stored at −80 °C. Each intestinal sample (100 mg) was individually macerated using a porcelain mortar and pestle and diluted to 20% (*w*/*v*) in 1 mL of phosphate-buffered saline (PBS) at pH 7.2. Throughout this procedure, the skin and mucous membranes of the captured animals were inspected for lesions suggestive of papillomatosis.

### 2.2. High-Throughput Sequencing

For each of the 22 vampire bats, 100 µL of the macerated intestine was collected and combined into a single pool. PBS (pH 7.2) was added to a final volume of 35 mL, which was then centrifuged at 10,000× *g* for 5 min at 4 °C. The supernatant was filtered through a 0.45 μm filter to remove debris, followed by ultracentrifugation at 150,000× *g* for 3 h in a 25% sucrose cushion. The pellet was resuspended in 1 mL of ultrapure water. DNA extraction was performed using magnetic beads with a MagMAX™ CORE nucleic acid purification kit (Applied Biosystems, Waltham, MA, EUA) according to the manufacturer’s instructions. The samples were evaluated for purity and quantity using a NanoDrop™ Lite Spectrophotometer (Thermo Fisher Scientific, Waltham, MA, USA) and a Qubit^®^ 2.0 fluorometer (Life Technologies, Waltham, MA, USA), respectively, and subjected to RCA using the Phi29 enzyme (New England—BioLabs, Ipswich, MA, USA) following the manufacturer’s instructions. The amplification product was purified with an Agencourt AMPure XP^TM^ Kit (Beckman Coulter Life Sciences, Indianapolis, IN, USA). The DNA libraries were further prepared with 1 ng of purified DNA using a Nextera XT DNA sample preparation kit and sequenced using a MiSeq Reagent Kit v2 300 (2 × 150 paired-end) on the Illumina^®^ MiSeq platform (San Diego, CA, USA).

### 2.3. Bioinformatic Analysis

The read quality was determined using FastQC software (version 0.12.0) through the OmicsBox platform (BioBam, 2019) [46]. Trimmomatic was used for adapters and quality trimming. The host genome was removed using Bowtie2. Assembly was performed using Meta-SPAdes, and the scaffolds were submitted to Diamond BLASTx. Open reading frame (ORF) prediction and genome annotation with EMBOSS 6.5.7 were performed with Geneious Prime software (version 2024.0.5).

Phylogenetic analyses were performed using W-IQ-TREE software [47]. The sequences were aligned using Multiple Alignment using Fast Fourier Transform (MAFFT) [48] and model selection was determined using ModelFinder [49]. Evolutionary history was inferred using the maximum likelihood method, with 1000 bootstrap replicates [50] for the L1 nucleotide tree and the L1, L2, E1, and E2 concatenated nucleotide sequence tree. Figtree v1.4.4 software was used for visualization [51]. The National Center for Biotechnology Information (NCBI) reference genomes for each genus were utilized for phylogenetic tree construction. The pairwise identities of the L1 gene matrices were calculated using MAFFT alignment with Geneious Prime software (version 2024.0.5). The sequences of the best hits were not included in the analysis, as they belong to unclassified and unpublished papillomaviruses.

Viral classification was performed following International Committee on Taxonomy of Viruses (ICTV) guidelines and the Papillomavirus Episteme website (PaVE) https://pave.niaid.nih.gov, (accessed on 10 September, 2025) [52], which is based on the nucleotide sequence identity of the L1 ORF, where species within the same genus must present more than 60% L1 identity, and PV types within a species share between 71% and 89% identity.

### 2.4. Papillomaviridae Conventional PCR Screening

To investigate the presence of PV genomes detected by HTS in the 22 individual intestine samples, specific primers for the L1 gene of each complete genome detected in this study were selected using Geneious Prime. The oligonucleotides and corresponding fragment sizes amplified are listed in Table 1. DNA isolation was performed using a standard phenol-chloroform protocol [53]. For PCR, Platinum™ Taq DNA Polymerase (Invitrogen, Waltham, MA, USA) was used, and the PCR was conducted in a total volume of 25 µL, containing 1x PCR buffer, 1.5 mM MgCl2, 0.2 mM deoxynucleotide triphosphates (dNTP), 0.2 µM forward primer, 0.2 µM reverse primer, Taq DNA polymerase (2 U), 2 µL of template DNA, and nuclease-free water up to the final volume. The PCR protocol conditions involved initial denaturation at 95 °C for 2 min, followed by 35 cycles of 95 °C for 30 s, 52 °C for 30 s, and 72 °C for 30 s. The amplification of a fragment of the glyceraldehyde-3-phosphate dehydrogenase (GAPDH) gene was used as an internal control [54]. Amplification products were sequenced using the Sanger method (ABI PRISM 3100 genetic analyzers; Big-Dye Terminator v.3.1 cycle sequencing kit (Thermo Fisher Scientific, Waltham, MA, USA). The sequences were assembled with Geneious Prime software (version 2024.0.5) and analyzed using BLASTx https://blast.ncbi.nlm.nih.gov/Blast.cgi (accessed on 03 September 2024).

## 3. Results

**Figure 2:**
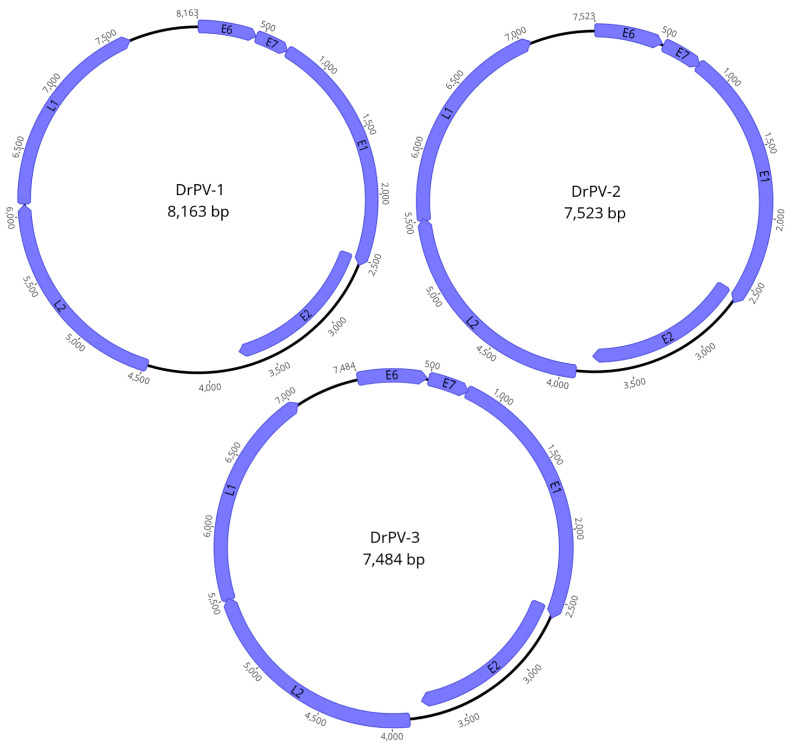
Schematic representation of DrPV-1, DrPV-2, and DrPV-3 genome organization. The numbers indicate the nucleotide sequence positions. ORFs E1, E2, E6, E7, L1, and L2 are represented. The first nucleotide of ORF E6 was assigned position number 1.

In the present study, a pool of 22 macerated intestine samples from *Desmodus rotundus* was subjected to high-throughput sequencing (HTS) on the Illumina MiSeq platform. In the HTS analysis, after trimming and host genome removal, a total of 2,969,804 reads were obtained, resulting in the assembly of 5763 contigs. After comparison with the NCBI nonredundant protein sequence (nr) database through Diamond BLASTx, three contigs of complete genomes related to the Papillomaviridae family were identified. The complete genome of each virus contained six Open Reading Frame (ORFs), four encoding early genes (E1, E2, E6, and E7) and two encoding late genes (L1 and L2), which encoded capsid proteins (Figure 2). Additionally, two noncoding regions were identified between the L1 and E6 genes (NCR1) and between L2 and E2 (NCR2). The putative three novel viruses were named Desmodus rotundus papillomavirus 1 (DrPV-1), Desmodus rotundus papillomavirus 2 (DrPV-2) and Desmodus rotundus papillomavirus 3 (DrPV-3), and their sequences were deposited in GenBank under the accession numbers PQ394573, PQ394574, and PQ394575, respectively.

Genomic information and BLASTX comparisons of the L1 ORF from DrPV-1, DrPV-2, and DrPV-3 are presented in Table 2. The best-hit viruses were not included in the phylogenetic analysis because they had not been classified into genera or species, and their sequences belonged to an unpublished article.

An extensive genome analysis of these three *D. rotundus* PVs revealed several amino acid and nucleotide motifs commonly found in papillomaviruses [32,33]. Two zinc-binding domains (CXXCX29CXXC) were present in all three papillomavirus E6 proteins and one in the E7 protein. The retinoblastoma protein-binding motif (pRB-binding motif) (LXCXE) was present in the E7 proteins of DrPV-1, DrPV-2, and DrPV-3, and the ATP-binding domain (GX4GKS) was found in all E1 proteins. The leucine zipper domain (LX6LX6LX6) was absent in each PV E2 protein. Polyadenylation signals (polyA) (AATAAA) were detected at distinct ORFs and NCRs of the three genomes described herein. One polyA stretch was found in the NCR of DrPV-3, whereas four polyA stretches were identified in the E1, E2, and E6 genes, as well as at NCR1 of the DrPV-2 genome. The genome of DrPV-1 encoded two polyA sites in the E2 gene and NCR1 and one at the L2 ORF. Two E2 binding sites (E2BS) (ACCG-N3/4/6-CGGT) were located in the NCR1 region and one in NCR2 of DrPV-1 nucleotide sequences. E2BS was absent in DrPV-2, while DrPV-3 presented this motif in the NCR1 region. Two E1 binding site (E1BS) [AT(A/G/T) G(C/T) (C/T)] were located in NCR1 and five in NCR2 of DrPV-1, whereas DrPV-3 presented one at the NCR1 and one in NCR2. Three E1BS were located at the NCR1 and one at NCR2 of DrPV-2. Two TATA box motifs were identified in NCR1 and NCR2 of DrPV-1. In contrast, the TATA box was missing in the DrPV-3 noncoding regions, whereas DrPV-2 presented two in the NCR2. A comprehensive description of the genomes, detailing the exact positions of the ORFs, amino acid motifs, and regulatory elements, is presented in Table S1. Although E4 ORFs could not be annotated in DrPV1, DrPV-2, or DrPV-3, there were characteristic proline-rich regions in the E2 ORF that may indicate the presence of E2^E4 [55] with proline percentages of 13%, 12%, and 15%, respectively.

**Figure 3:**
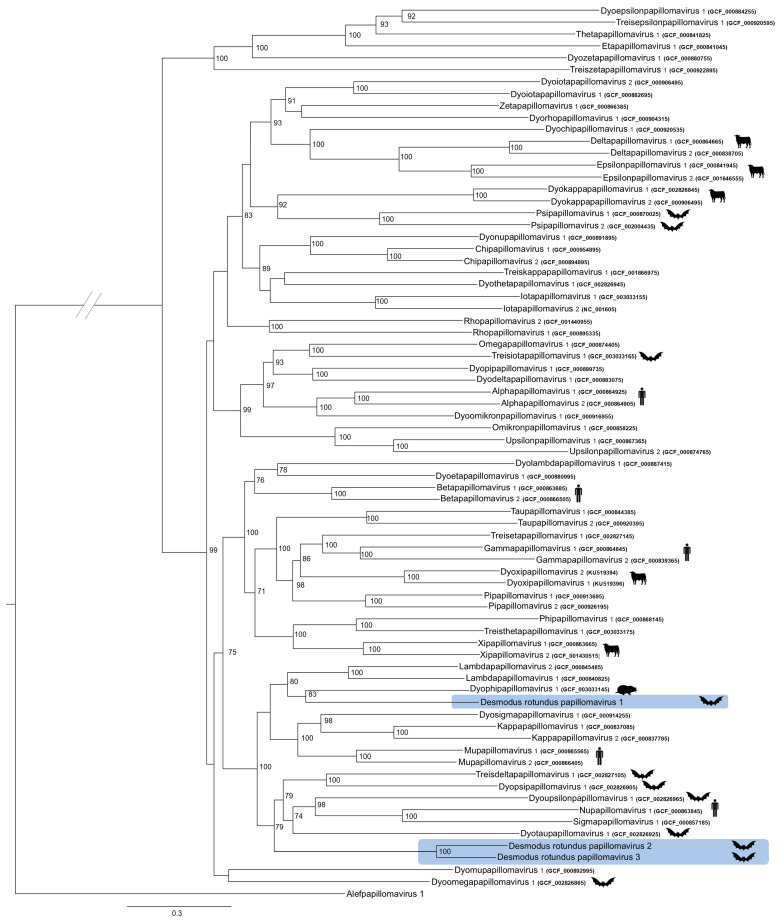
Phylogenetic analysis of species from each papillomavirus genus. Concatenated E1, E2, L1, and L2 nucleotide sequences. A maximum likelihood tree was produced using the substitution model (GTR + F + I + G4). Nodes supported by >70% bootstrap replicates are indicated. The diagonal lines on the branches indicate that the evolutionary distance is greater than visually represented.

Phylogenetic trees were constructed based on concatenated E1-E2-L1-L2 genes and L1 individual genes using the maximum likelihood approach at the nucleotide level. Both approaches yielded similar results (Figure 3 and Figure S2). In the two phylogenetic trees, DrPV-1 and Dyophipapillomavirus 1, a virus species isolated from the European mole (*Talpa europaea*), were grouped into a clade with bootstrap values of 83 and 88 for the concatenated and L1 sequence trees, respectively. The L1 pairwise identity matrix revealed that DrPV-1 shared 60.04% identity with *Dyophipapillomavirus* 1 (Table S2), whereas DrPV-1 also displayed L1 identity percentages of approximately 60% with other genera, including Lambdapillomavirus 1 (58.87%), Lambdapapillomavirus 2 (60.13%), Dyopsipapillomavirus 1 (58.99%), Dyosigmapapillomavirus 1 (59.39%), and Treisdeltapapillomavirus 1 (61.03%). At L1 gene and concatenated E1-E2-L1-L2 trees, DrPV-2 and DrPV-3 clustered into a distinct clade, representing a new PV genus. These two genomes shared an L1 identity of 73.47%; therefore, they can be classified as different PV types belonging to the same species.

To identify which of the 22 bats carried the viruses detected through Illumina sequencing, new oligonucleotides were designed based on the L1 gene of each papillomavirus genome (Table 1). DrPV-1 was successfully detected in the intestine of *D. rotundus* bat number 14, with 342 bp of the L1 fragment (GenBank ID PQ356734). Despite several attempts, DrPV-2 and DrPV-3 could not be amplified from the intestinal samples of individual bats. The internal control GAPDH gene fragment was amplified from all intestine samples.

## 4. Discussion

Bats are animals that host many pathogens, including both zoonotic and non-zoonotic viruses. The analysis and study of the chiropteran unbiased virome through the implementation of surveillance through sequencing are of utmost importance, primarily in a One Health context [56]. High-throughput sequencing (HTS), along with corresponding progress in bioinformatic tools, has become an essential methodology in many science fields, used for multiple purposes. The metagenomic era has not only enhanced our understanding of viruses but also led to the discovery of several new viral agents, including new species, genera, and even entire viral families. This has significantly expanded our knowledge of the virosphere, contributing to viral taxonomic classification as we know it today [57].

Herein, we describe the detection and genetic description of three novel *D. rotundus* PVs (DrPV-1, DrPV-2, and DrPV-3), which demonstrated typical PV genome organization, with six ORFs (E1, E2, E6, E7, L1, L2) and two noncoding regions (NCR1 and NCR2). The DrPV-1 genome contained a long NCR2 between L2 and E2, a feature also found in distantly related bat papillomaviruses, a region that may have arisen from distinct, independent recombination events during their evolutionary history [33]. Papillomavirus infections frequently lead to the development of benign or malignant neoplastic tumors in the mucous and cutaneous epithelia of a variety of vertebrate species [12,13,14], such as bats [58]. The first bat PV described in the literature was isolated from a basosquamous carcinoma in an Egyptian fruit bat (*Rousettus aegyptiacus*) [58]. However, despite the detection of three different PVs in the intestine/feces of *D. rotundus* in this study, no mucosal or skin lesions were noted on the animals. PVs are known to be detected in healthy host tissues, without clinical signs, which includes animals and humans, such as some types of bovine papillomavirus (BPV) [59,60] and avian PVs [61], as well as HPVs [62]. Bat PVs have been found mainly in the healthy lower gastrointestinal tract, including the intestinal, rectal, and fecal samples of many chiropterans species [32,33,34,35,36,37,38,39,40,41,42,43,44].

In the individual bat sample PV screening, carried out using conventional PCR, it was possible to identify part of the L1 gene of DrPV-1 in the intestine of *D. rotundus* number 14 (GenBank ID PQ356734). This male bat was collected under a bridge in the municipality of Santana do Livramento, RS, in August 2022. Although several attempts were made, it was not possible to identify DrPV-2 and DrPV-3 in any of the 22 bats, corroborating other studies that faced the same challenge [32,37]. Some possible explanations are the low viral load in the original sample before enrichment (RCA) and the loss of integrity of the genetic material, as PCR requires intact sequences for DNA amplification.

Strictly based on the nucleotide sequence of L1, DrPV-1 could be classified as a new species within the *Treisdeltapapillomavirus* genus. This genus currently consists of a single bat PV species isolated from *Rhinolophus ferrumequinum* (GenBank ID: GCF_002827105.1), with which DrPV-1 shared 61.03% L1 identity. Although, the genome described herein also shared more than 60% L1 identity with *Lambdapillomavirus* 2 (Lambdapillomavirus genus) isolated from a canine oral papillomavirus (GenBank ID: GCF_000840825). However, beyond L1 nucleotide identity, analyzing the virus’s topological position within the L1 phylogenetic tree as well as the concatenated nucleotide (E1, E1, L1 and L2) phylogenetic tree [11,63] is recommended by ICTV. Considering the concatenated tree (Figure 3), DrPV-1 was evolutionarily closely related to a virus species isolated from *Talpa europaea*, named TePV-1 (GenBank ID GCF_003033145); hence, DrPV-1 could represent a novel species of the *Dyophipapillomavirus* genus. Interestingly, both DrPV-1 and TePV-1 had long NCR2 sequences [64]. The phylogenetic clustering also indicated that DrPV-1 shared common ancestry with the *Lambdapapillomavirus* genus, as they belong to the same clade, which is later divided into two monophyletic clades, *Lambda* and *Dyophi* papillomaviruses.

DrPV-2 and DrPV-3 did not share more than 60% L1 nucleotide identity with any other existing genera. DrPV-2 shared 56.22% nucleotide identity with *Kappapapillomavirus* 1 (GenBank ID GCF_000837085), a virus isolated from a cottontail rabbit, and DrPV-3 shared 56.85% nucleotide identity with *Mupapillomavirus* 2 (GenBank ID GCF_000865565), a human papillomavirus type 63. DrPV-2 and DrPV-3 shared 73.47% L1 identity, indicating that they belong to the same species within a new genus. As shown in Figure 3, they cluster within the same monophyletic clade, reaffirming that they belong to a novel genus. PV types within a species share between 71% and 89% nucleotide identity within the complete L1 ORF [65]. These results indicate that DrPV-2 and DrPV-3 are different types of the same species from a putative novel genus.

In a recent publication, the classification of PVs was revisited and discussed. The 60% L1 identity threshold, which was implemented in 2004 [65], was based on PV diversity at that time. However, this classification was biased by the *Alphapapillomavirus* genus, as 59 of the 118 viruses used in the previous analysis belonged to that genus [63]. Given this, the 60% threshold does not represent the diversity of papillomaviruses now available in databases and may not work for all *Papillomaviridae* members. Therefore, the existing classification system has limitations that must be further addressed. To the best of the authors’ knowledge, herein, we present the first report of a papillomavirus from the *Desmodus rotundus* species, adding to the characterization of PVs from understudied taxonomic groups, such as bats. Furthermore, studies in this field are crucial for shedding light on the evolutionary processes that drive PV diversification.

## 5. Conclusions

This study reports the first identification of PVs in the *Desmodus rotundus* species, enhancing the understanding of papillomavirus diversity in bats. The discovery of three novel PVs (DrPV-1, DrPV-2, and DrPV-3) through RCA enrichment followed by HTS resulted in the identification of a novel genus with two types of the same species and a putative novel species within the *Dyophipapillomavirus* genus, contributing to the characterization of PVs in the Chiropteran order.

## Figures and Tables

**Table 1:** DrPV-1, DrPV-2, and DrPV-3 L1-specific oligonucleotide sequences.

*D. rotundus* PVs	Primer Sequence	Product Size	Tm
DrPV-1 F	5′ TTGCTCACAGTGGGTCATCC 3′	421 nt	60 °C
DrPV-1 R	5′ CTGGCTCTTCTCCAGCACAA 3′		55 °C
DrPV-2 F	5′ TCTGTGGCTGGCAATCCATT 3′	496 nt	60 °C
DrPV-2 R	5′ TACCACGGGCCATATCCTCA 3′		60.1 °C
DrPV-3 F	5′ TTGCCCGCCATTTCTTTGTG 3′	494 nt	60 °C
DrPV-3 R	5′ CAGAGTGTCAGTTGGGGGAC 3′		60 °C

**Table 2:** Genomic data and BLASTX comparison of three *Desmodus rotundus* papillomaviruses, highlighting genome size, Guanine-Cytosine (GC) content, coverage, and similarity with reference viruses.

Virus	Genome Length	CG%	Coverage (Reads)	BLASTX Best Hit: L1% Identity	Query Cover (%)
DrPV-1	8165 bp	43.1	3968	*Rhinolophus bat papillomavirus* 1 (WXG28025.1) 66.18%	92
DrPV-2	7579 bp	44.7	2165	*Taphozous bat papillomavirus* 1 (ID: WXG28296.1) 60.56%	94
DrPV-3	7561 bp	43.9	5549	*Taphozous bat papillomavirus* 1 (ID: WXG28300.1) 61.19%	97

## Data Availability

All data generated or analyzed during this study are included in this published article and its Supplementary Information Files. The complete genome sequences and the L1 gene fragment data from this study have been deposited in the NCBI GenBank database under the accession codes PQ394573, PQ394574, PQ394575, and PQ356734.

## Supplementary Materials

The following supporting information can be downloaded at: https://www.mdpi.com/article/10.3390/ani14243604/s1, Figure S1: Phylogenetic analysis of species from each papillomavirus genus. L1 nucleotide sequences. A maximum likelihood tree was constructed using the substitution model (GTR+F+I+G4). Nodes supported by >70% bootstrap replicates are indicated. The diagonal lines on the branches indicate that the evolutionary distance is greater than visually represented; Table S1: Motif annotation of DrPV-1, DrPV-2, and DrPV-3; Table S2: Pairwise identity percentage matrix of L1 nucleotides.
