# Supplementary material for: Characterization of Three Novel Papillomavirus Genomes in Vampire Bats (Desmodus rotundus)
Source: Animals (Basel). 2024 Dec 14;14(24):3604. doi: 10.3390/ani14243604 (PMC11672418; doi:10.3390/ani14243604)
Supplement: Supplementary file 1 [file animals-14-03604-s001.zip › Figure S1 caption.pdf]

Figure S1: Phylogenetic analysis of species from each papillomavirus genus. L1 nucleotide sequences. A maximum likelihood tree was constructed using the substitution model (GTR+F+I+G4). Nodes supported by >70% bootstrap replicates are indicated. The diagonal lines on the branches indicate that the evolutionary distance is greater than visually represented.
