# Supplementary figures and images for: Characterization of Three Novel Papillomavirus Genomes in Vampire Bats (Desmodus rotundus)
Source: Animals (Basel). 2024 Dec 14;14(24):3604. doi: 10.3390/ani14243604 (PMC11672418; doi:10.3390/ani14243604)

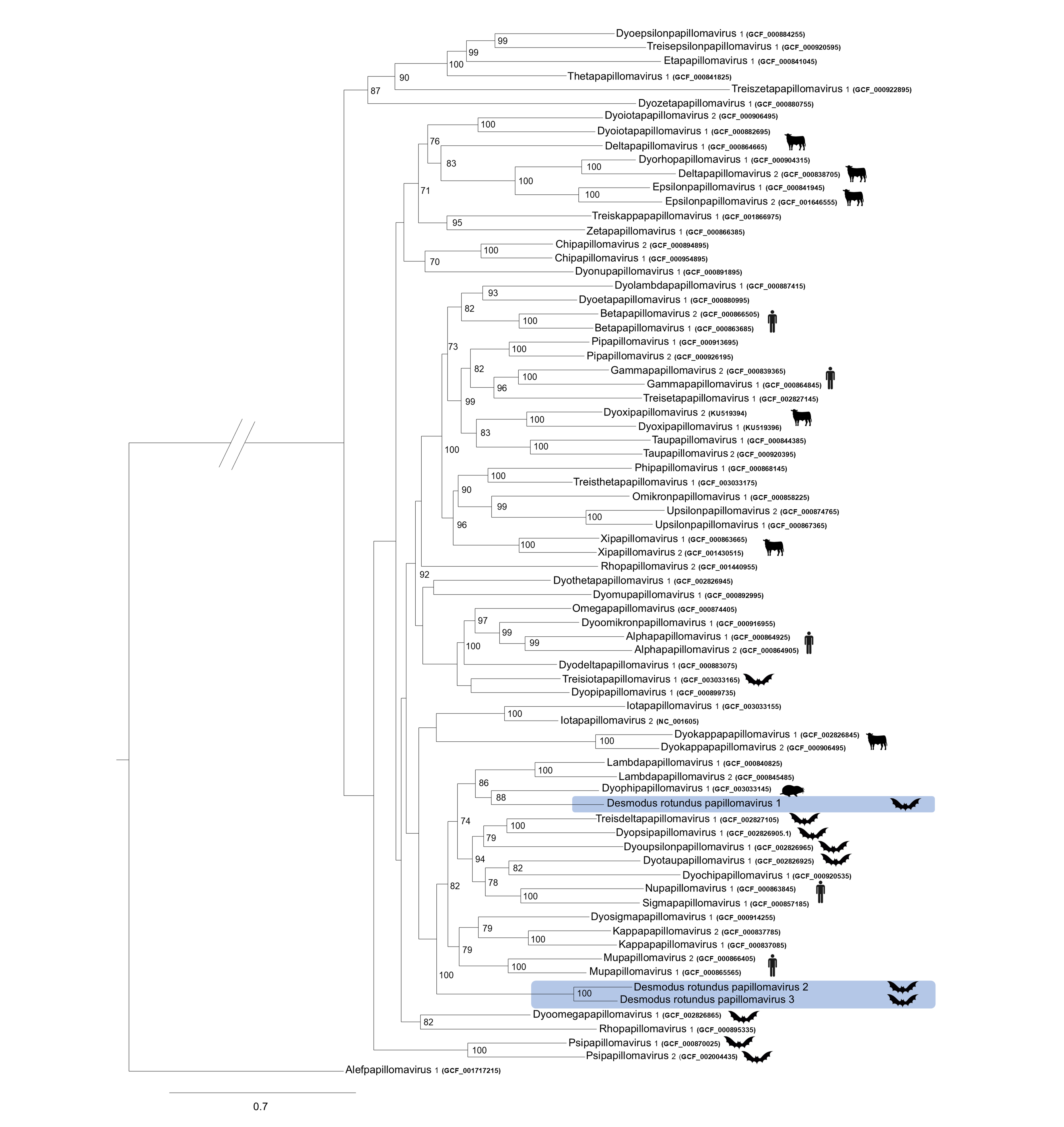

Supplement: Supplementary file 1 [file animals-14-03604-s001.zip › figure S1 L1 tree.tif]
